# A hybrid approach of ensemble learning and grey wolf optimizer for DNA splice junction prediction

**DOI:** 10.1371/journal.pone.0310698

**Published:** 2024-09-23

**Authors:** Eslam Hamouda, Mayada Tarek

**Affiliations:** 1 Computer Science Department, Faculty of Computers & Information, Mansoura University, Mansoura, Egypt; 2 Computer Science Department, Faculty of Computers & Information, Jouf University, Jouf, Saudi Arabi; Nottingham Trent University School of Science and Technology, UNITED KINGDOM OF GREAT BRITAIN AND NORTHERN IRELAND

## Abstract

DNA splice junction classification is a crucial job in computational biology. The challenge is to predict the junction type (IE, EI, or N) from a given DNA sequence. Predicting junction type is crucial for understanding gene expression patterns, disease causes, splicing regulation, and gene structure. The location of the regions where exons are joined, and introns are removed during RNA splicing is very difficult to determine because no universal rule guides this process. This study presents a two-layer hybrid approach inspired by ensemble learning to overcome this challenge. The first layer applies the grey wolf optimizer (GWO) for feature selection. GWO’s exploration ability allows it to efficiently search a vast feature space, while its exploitation ability refines promising areas, thus leading to a more reliable feature selection. The selected features are then fed into the second layer, which employs a classification model trained on the retrieved features. Using cross-validation, the proposed method divides the DNA splice junction dataset into training and test sets, allowing for a thorough examination of the classifier’s generalization ability. The ensemble model is trained on various partitions of the training set and tested on the remaining held-out fold. This process is performed for each fold, comprehensively evaluating the classifier’s performance. We tested our method using the StatLog DNA dataset. Compared to various machine learning models for DNA splice junction prediction, the proposed GWO+SVM ensemble method achieved an accuracy of 96%. This finding suggests that the proposed ensemble hybrid approach is promising for DNA splice junction classification. The implementation code for the proposed approach is available at https://github.com/EFHamouda/DNA-splice-junction-prediction.

## Introduction

**Fig 1:**
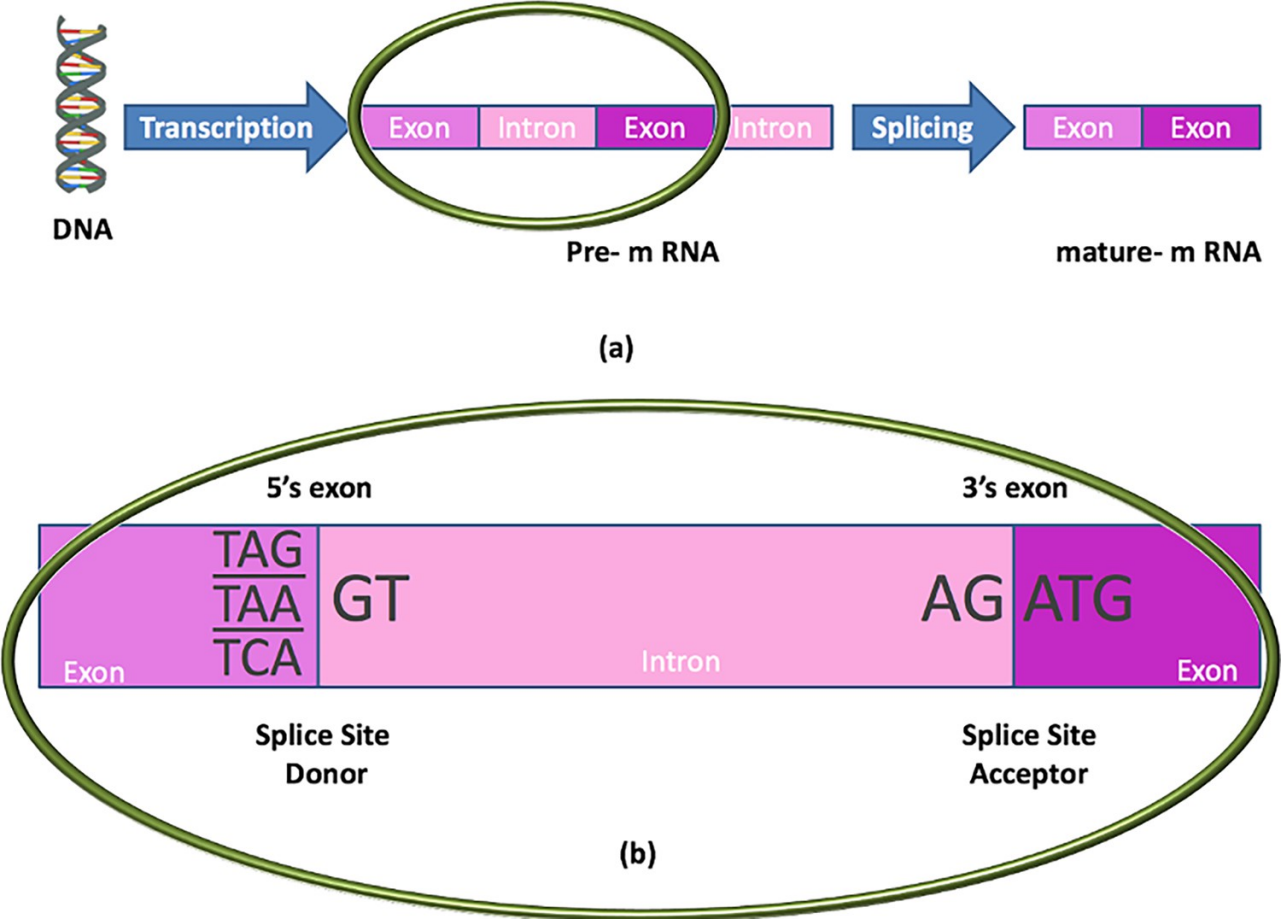
DNA Splicing process (a), Pre-m RNA inside look (b).

DNA, a biological molecule, acts as a template for the creation of an organism. Its structure comprises two strands of nucleotides joined by complementary base pairs. These nucleotides, marked by A, G, C, and T, are adenine, guanine, cytosine, and thymine [1]. Pre-mRNA is an intermediary form of DNA that includes genetic information but differs from DNA in its single-stranded structure and different nucleotide makeup. Pre-mRNA contains coding sequences called exons and non-coding sequences called introns, with at least one intron region between two exons, as shown in Fig 1(A). RNA splicing is a critical step in gene expression. The process entails removing introns from pre-mRNA and combining exons to generate mature mRNA. Splice junctions, the borders between exons and introns, are essential in this process. They act as boundary markers between these two sections. DNA splice junction prediction is the procedure for recognizing these boundaries during splicing. As depicted in Fig 1(B), splice sites usually consist of four conserved nucleotides: the donor sequence GT (GU for pre-mRNA) at the 5’ end (at the exon-intron boundaries) and the acceptor sequence AG at the 3’ end (at the intron-exon boundaries). In the coding sequence, introns begin with GT bases and conclude with AG bases, whereas exons begin with ATG and end with one of three stop codons: TAG, TAA, or TCA [2].

The length of splice junctions usually is 20–30 nucleotides that contain specific motifs recognized by the splicing machinery. The number of splice junctions varies in a single DNA sequence, depending on the gene’s length and complexity. However, a single DNA sequence has 10–50 splice junctions [3]. The splice junctions’ sequences can be classified into three classes: IE, EI, or N:

- IE boundary: occurs when the intron starts with an intron splice site (ISS) and the exon ends with an exon splice site (ESS). The consensus sequence for an IE boundary is MAG|GUR, where M is A or C, A is A or G, G is G, U is U or T, and R is A or G.
- EI boundary: occurs when the exon starts with an ESS and the intron ends with an ISS. The consensus sequence for an EI exon boundary is YUR|NAG, where Y is C or T, U is U or T, R is A or G, N is A, C, G, or T, A is A or G, and G is G.
- N boundary: occurs when the exon starts with an ESS and the intron ends with a non-splice site. The consensus sequence for an N boundary is YUR|NNN, where Y is C or T, U is U or T, R is A or G, and N is A, C, G, or T.

Knowing the boundary type (IE, EI, or N) is crucial for understanding gene expression patterns, disease causes, splicing regulation, and gene structure. Its applications in biology and medicine range from drug discovery and gene prediction to comprehending intricate biological processes and creating personalized health plans [4]. Because no general rule governs this process, the exact location where exons are connected, and introns are deleted during RNA splicing poses a substantial challenge. Machine learning methods are an effective and powerful tool for predicting splice sites. Support Vector Machines (SVMs), artificial neural networks (ANNs), and Random Forest (RF) algorithms have all made significant contributions to this area [5–7].

Moreover, Bayesian networks and Maximum Entropy Distribution (MED) models have also been applied for boundary prediction [8, 9]. The use of machine learning for DNA splice junction classification faces numerous challenges. First, the high dimensionality of DNA sequences makes identifying relevant features and training practical classification algorithms difficult. Second, noise and errors in sequencing data may impair classification model performance and result in inaccurate predictions. Eventually, existing methods may overfit the training data over time, resulting in poor generalization performance on unseen data.

Given the previous challenges, this paper provides a new approach to predicting DNA splice junctions. The suggested approach consists of two layers and is based on a combination of feature selection and ensemble learning techniques. The primary objective of GWO-based feature selection is to identify the most relevant features from high-dimensional data. Due to its exploration and exploitation capabilities, GWO finds relevant features through a larger feature space. On the other hand, the ensemble learning approach improves the generalization ability of the classification model. The remaining sections of this article are organized as follows: The Related works section summarizes the relevant research works for DNA splice junction prediction, including deep learning techniques and a review of metaheuristic algorithms and their applicability to the DNA problem. The Methodology section describes the proposed hybrid approach of grey wolf optimizer and ensemble learning for DNA splice junction prediction. The Experiments section introduces the experiments and performance evaluation. The Discussion section shows the impact of the findings on the proposed DNA splice junction prediction approach. Finally, the article is concluded in the Conclusion section.

## Related works

### Deep learning algorithms for DNA junction predication

Several deep-learning architectures have shown significant promise in predicting splice junctions. Convolutional Neural Networks (CNNs) have been particularly successful. For instance, a simple CNN-based approach achieved 96% accuracy in splice junction recognition [10]. Building on this success, studies like SpliceFinder [11] utilized CNNs for classification and achieved an accuracy of 90.25%. Additionally, research has explored Recurrent Neural Networks (RNNs) for splice junction prediction. One study employed a Bidirectional Long Short-Term Memory (BLSTM) network on a GENCODE-derived dataset [12]. Beyond these initial studies, further research focuses on CNN and RNN architectures. DeepSplice [13] introduced a high-performance CNN, while studies like REIDS [14] and ASJA [15] explored alternative approaches for identifying splicing types using linear mixed models and sophisticated data analysis pipelines. Additionally, LSTM models have been developed to predict exon inclusion based on epigenetic signals [16]. It is important to note that the success of these deep learning models often depends on the size and quality of the training data. Studies like EDeepSSP [17], Splice2Deep [18], and InterSSPP [19] all demonstrate high performance in splice site classification tasks, likely due to being trained on large datasets. This highlights the importance of utilizing extensive training data for robust and accurate splice junction prediction using deep learning.

Furthermore, examining existing techniques highlights their varying efficacy across different splice junction types, necessitating the continued exploration of novel methods for splice site identification. Convolutional Neural Networks (CNNs) are wildly successful in scenarios when data displays intrinsic local correlations [20]. Recurrent neural networks, RNNs, are often a strong choice for analyzing time-series data [21]. However, RNNs have certain drawbacks, namely the need for pre-segmented training data. Long Short-Term Memory (LSTM) networks have effectively overcome these constraints, showing impressive performance for splice junction prediction [22]. Bidirectional LSTM (BLSTM) has been used to further improve splice junction prediction performance. To obtain a more comprehensive context and extract more subtle information from the sequence, BLSTMs process data in both forward and reverse directions [4, 23]. Recently, a study that compares CNN, BGRU, and BLSTM models for RNA splice site prediction has been presented [24]. Table 1 summarizes the recent techniques that have been noticed for their contributions to DNA splice junction prediction.

**Table 1:** Recent DNA splice junction prediction approaches.

Reference	Year	Technique	Method	Data	Performance
Zudlaret et al. [10]	2018	CNN	SpliceRover	NN269	96.12% (accuracy)
Wang et al. [11]	2019		SpliceFinder	Ensembl	Up to 90.25% (accuracy)
Zhang et al. [13]	2018		DeepSplice	HS3D	0.983 (AUC)
Amilpur et al. [17]	2020		EDeepSSP	HS3D	0.9870 (AUC)
Albaradei et al. [18]	2020		Splice2Deep	Homo sapiens	96.91% (F-measure)
Dasari et al. [19]	2020		InterSSPP	HS3D / NN269	0.9946 / 0.9922 (AUC)
Van et al. [14]	2018	Linear mixed models	REIDS	HJAY	65–77% (accuracy)
Zhao J et al. [15]	2019	Assembling Splice Junctions Analysis	ASJA	GEO	91.5%. (Sensitivity)
Lee et al. [16]	2020	LSTM and Gated Recurrent Unit	GRU	ENCODE Consortium	Up to 86% (F-measure)
Zabardast et al. [24]	2023	CNN / BLSM / BGRU		HS3D / C. elegans	92% / 96% (accuracy)

### Meta-heuristic optimization algorithms in DNA

Metaheuristic algorithms are problem-solving techniques that use nature-inspired approaches to find optimal solutions without requiring complex mathematical models [25]. Examples include genetic algorithms, swarm intelligence, and grey wolf optimizer. These algorithms have been successfully applied to various optimization problems in the field of bioinformatics. A multi-objective artificial fish swarm algorithm (MOAFS) has been developed to optimize multiple sequence alignment, using two fitness functions to ensure alignment quality and consistency [26]. A novel hybrid model known as PSOSA has been developed for solving multiple sequence alignment, integrating particle swarm optimization (PSO) for global search and simulated annealing (SA) for local optimization [27]. A novel discrete PSO algorithm has been introduced that operates directly within the search space, incorporating heuristic information and/or local search strategies [28]. A modified crow search algorithm (CSA) has been proposed to address the DNA fragment assembly problem, integrating CSA with a local search technique [28]. A novel analytical technique for SBH has been developed by integrating hypergraph with a genetic algorithm (HGGA) to extract DNA sequences from their spectra [29]. A novel algorithm, PSORPS, has been developed for identifying DNA motifs, it utilizes a random projection strategy to filter out noisy subsequences and construct the objective space, the predominant DNA sequence segments are then extracted to initialize the PSO population [30]. Table 2 summarizes the applications of metaheuristic algorithms for solving various DNA-related problems.

**Table 2:** Metaheuristic algorithms for solving DNA-related problems.

Reference	Year	Problem	*Metaheuristic* Technique
Dabba et al [26]	2019	Multiple sequence Alignment	Artificial fish swarm
Chaabane [27]	2018		PSO & SA
Ali et al. [28]	2020	DNA fragment assembly problem	PSO
Allaoui et al. [28]	2018		Hybrid crow Search algorithm
Swaminathan et al. [29]	2019	DNA *Sequencing by hybridization*	Hypergraph-based GA
Ge et al. [30]	2019	Finding motifs in DNA	Random projection and PSO

## Methodology

**Fig 2:**
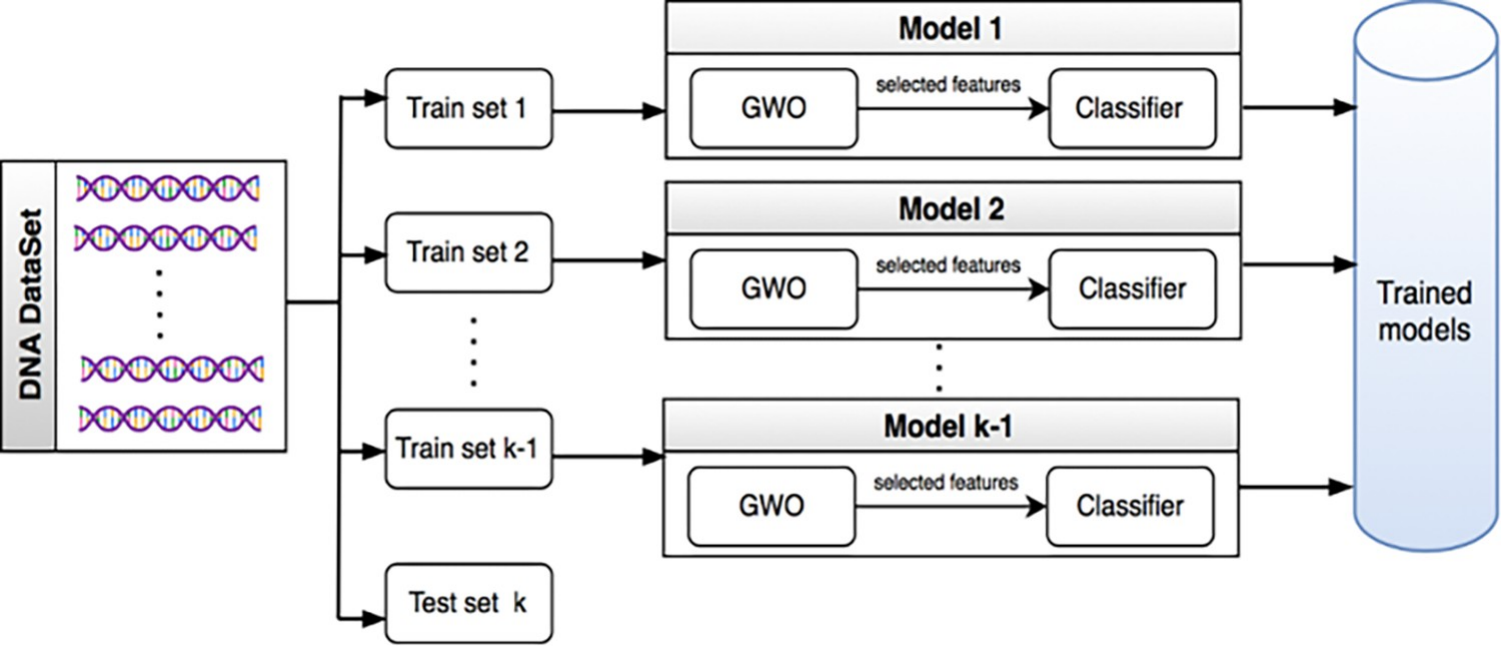
The architecture diagram for the proposed ensemble-hybrid approach.

The computational biology task of DNA splice junction classification involves predicting the junction type (IE, EI, or N) from a given DNA sequence. This study employs a two-layer hybrid approach inspired by ensemble learning to tackle this challenge. The grey wolf optimizer (GWO) [31] is employed in the first layer for feature selection. It is essential to recognize the significant traits while discarding irrelevant ones successfully. Subsequently, the second layer employs a classification model based on the features selected by the previous layer. The type of DNA splice junction is predicted from a given lengthy DNA sequence. The proposed ensemble learning approach combines GWO and the ensemble classifier to predict DNA splice junction types. The role of GWO is to provide the key features which enhance classification accuracy. Besides, unlike existing methods that may overfit the training data and perform poorly on unseen data, the proposed ensemble-hybrid method utilizes the power of ensemble learning. Combining predictions from multiple models trained on diverse feature subsets reduces the variance and improves the generalizability of the classifier, thus making the performance more robust on unseen DNA sequences. The architectural diagram of the proposed method is shown in Fig 2.

The suggested approach uses cross-validation to partition the DNA splice junction dataset into training and test sets, as seen in the figure. The ensemble models are trained on a variety of training set partitions. The ensemble’s performance is subsequently evaluated based on the remaining set, the held-out fold. This process is repeated for each split to maintain a comprehensive assessment of the classifier’s ability to generalize to previously unseen data. The pseudocode for the proposed ensemble approach for DNA splice junction prediction is provided in Algorithm 1.

Algorithm 1: The proposed ensemble approach

**Input:** DNA splice junction sequences (*S*), fold size (k).

**Output:** The overall classification performance.

 **1** Split the splice junction sequences (*S*) into disjoints equal folds: *S*_1_, *S*_2_,….,*S*_*k*_.

 **2** Set h = k, the index of the initial held-out test fold.

 **3 DO**

 **4** Initialize empty training models set, M→*ϕ*.

 **5**  **For *i* =** 1 to *k*

 **6**   **If** (i = = *h*), skip this iteration.

 **7**   Create an ensemble model *M*_*i*_ for fold *S*_*i*_ using the steps described in Algorithm 2.

 **8**   Set M →{*M*∪*M*_*i*_}.

 **9**  **End**

 **10** Retrieve the trained ensemble models set, M.

 **11**  **For each** ensemble model, *M*_*j*_ ∈ M

 **12**   Test the trained ensemble model *M*_*j*_ using sequences in the test fold *S*_*h*_.

 **13**   Set *d*_j_ to the class labels produced by *M*_*j*_.

 **14**  **End**

 **15** Apply majority voting among all *d*_*j*_ to set the final class labels.

 **16** Compute the classification performance for the current split using metrics specified by Eqs (3–8).

 **17** Set h = h-1.

 **18 While** (h>0).

 **19** Return the average classification performance among all splits.

**Fig 3:**
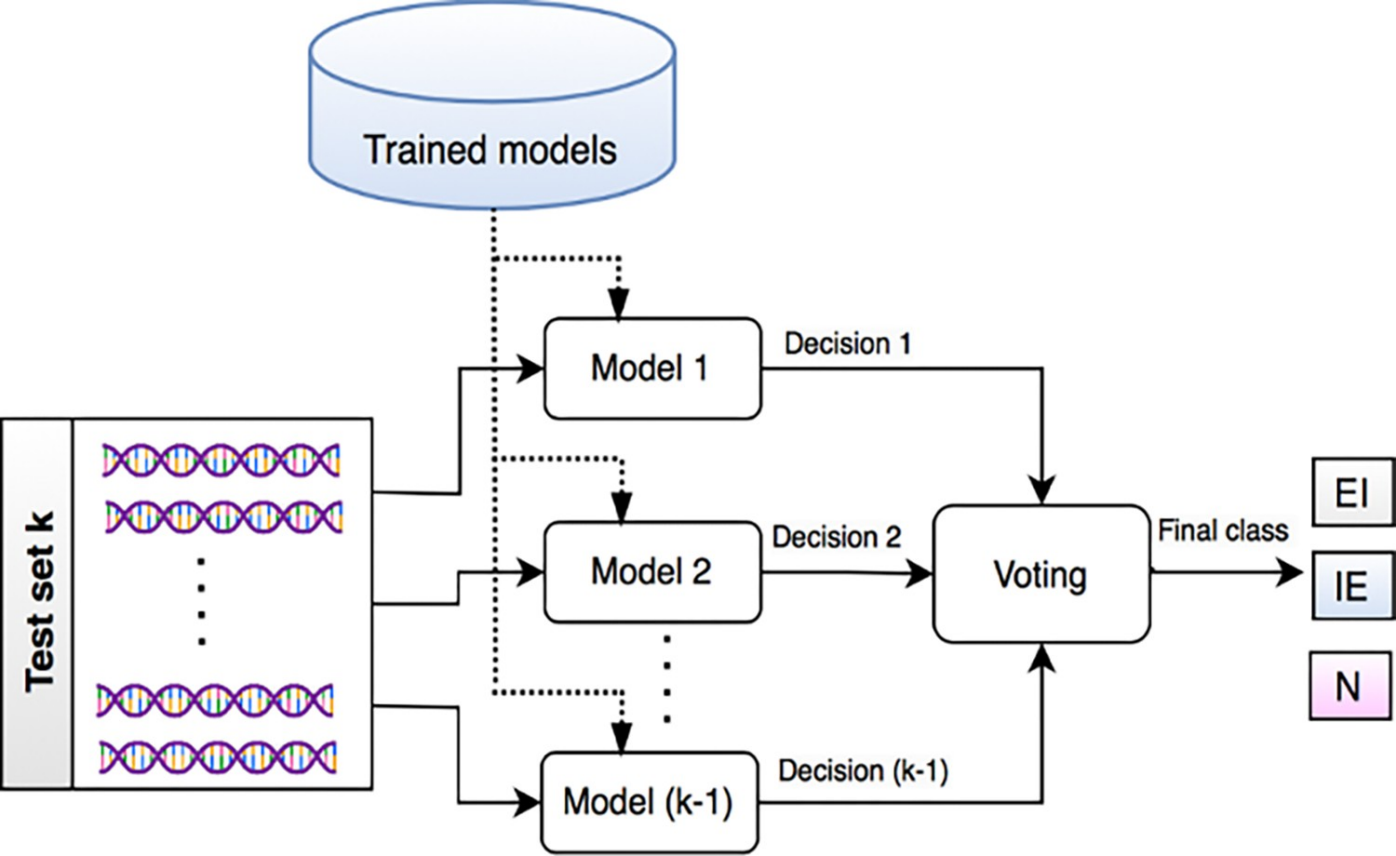
Testing phase for the proposed ensemble-hybrid approach.

The first layer of each ensemble model employs GWO to find the most relevant features from the training data. The optimization process is formulated as a minimization problem using a fitness function representing the classification error obtained using different feature subsets. Meanwhile, the second layer trains an ensemble machine-learning model using the selected features generated by the first layer. Ensembles are a powerful technique for improving classification accuracy since they combine the predictions of many models. Furthermore, it helps to reduce the risk of overfitting and improve the generalization performance of the classifier [32]. Upon successful training, the ensemble model is stored for subsequent classification of new DNA splice junctions by determining the most likely junction type based on the selected features. Fig 3 depicts the testing approach for the suggested hybrid method.

During the testing phase, the trained models are used to predict the junction type for the given DNA sequence, and the final decision is made using majority voting. The following subsections describe the layers of each ensemble model in more detail.

### Feature selection layer

GWO is a bio-inspired metaheuristic algorithm inspired by the intricate social hierarchy and sophisticated hunting behavior of grey wolves in nature. In this layer, GWO is designed to identify the optimal subset of features that maximizes the classification accuracy. Incorporating a chaotic map to update the GWO’s control parameter can effectively explore the feature space while exploiting promising feature subsets, enabling it to identify the most relevant features. The steps applied by this layer are listed below:

1. Create an initial random population of wolves to represent potential feature subsets. Each wolf is encoded as a binary vector, with each bit representing the presence or absence of a given feature (0 representing exclusion, 1 representing inclusion).
2. Calculate the fitness of each wolf in the population using a classification model trained on the selected features.
3. Identify the positions of alpha, beta, and delta wolves. The alpha, beta, and delta wolves represent the best, second, and third feature subsets.
4. Update the control parameter a, using one of the chaotic maps presented in [33].
5. Update the positions of wolves using the update functions presented in the original GWO algorithm [31].
6. Apply the following threshold function to each element of the wolves’ position to maintain a binary representing, represent the selected features subset.
$$w_{i}=\left\{ \begin{array}{l} 1,\;if\;(w_{i}\geq 0.5)\\ 0,\;otherwise \end{array}\right.$$ (1)
  This equation applies a threshold function to each element of "wolves" arrays (each wolf represents a candidate feature subset in the GWO algorithm). The threshold function transforms the value of each element into a binary representation. This binary form assists in deciding whether a feature is part of the final selected feature subset needed for the DNA splice junction prediction.
7. Repeat steps 2–5 until a maximum number of iterations is reached.
8. Select the features corresponding to the bits set to 1 in the alpha wolf’s position; this subset represents the most relevant features for the classification.

### Classification training layer

The role of this layer is to train a classification model using the features selected by the previous layer. This trained model is used as the fitness function to evaluate each wolf’s efficiency in the population. The dataset employed in this layer is partitioned into a training set, utilized for training the classifier, and a validation set, employed by the fitness function, to assess the efficacy of the candidate solution. The objective function presented by this layer is calculated as follows:

$$F(w)=\frac{\sum_{i=1}^{n}\left(P_{i}\neq A_{i}\right)}{n}$$ (2)

This equation defines the objective function used within the GWO framework for DNA splice junction prediction. The objective function represents the fitness of a particular candidate feature subset (represented by a "wolf"). It considers w, n, Pi_,_ and A_i_ to evaluate a feature subset’s performance, where *w* represents the set of features selected by the wolf. P_i_ and A_i_ denote the predicated and the actual class labels generated by the classifier using the selected features set, respectively, and *n* denotes the number of DNA sequences in the validation set. A lower objective function value indicates a better-performing feature subset for predicting the type of DNA splice junctions.

In this study, four distinct classifiers were employed: support vector machines (SVM), decision trees (DT), k-nearest neighbour (KNN), and Naïve Bayes classifier (NB).

Support vector machine (SVM) is a widely used machine learning algorithm for classification tasks. SVM is initially presented for binary classification problems [34]. SVM is extended to use in the DNA splice junction multi-class problem using the one-against-one approach. The proposed methodology entails training binary SVM classifiers for each pair of classes. To classify a new DNA sequence, the proposed method conducts a majority vote among the binary classifiers, assigning the class label that receives the highest number of votes.

Decision trees (DT) are a supervised learning algorithm for classification and regression tasks. DTs are constructed through recursive partitioning steps that divide the data into smaller subsets based on predefined decision rules [35]. The DT algorithm starts with a single node containing all the training DNA sequences. Subsequently, the algorithm recursively partitions the data into increasingly smaller subsets until each subset solely comprises data points possessing the same classification label. The decision rules governing each partition are determined by the information gain accrued from the respective split. Upon construction, it is employed to classify the unseen DNA sequence.

The k-nearest neighbors (KNN) algorithm is an instance-based learning algorithm for classification and regression. The idea of the algorithm is based on the concept that similar data points are likely to have similar labels [36]. The distances between the DNA sequence and those in the training set are computed using the Hamming distance metric to predict the junction type for an unseen DNA sequence. Subsequently, distances are arranged in ascending order, and the five nearest neighbors are selected. Eventually, the class label is assigned based on the most frequent class label among the selected nearest neighbours.

The Naive Bayes is a valuable supervised learning algorithm for multi-class classification problems [37]. It is relatively easy to implement and incorporate due to its simplicity. The Naive Bayes algorithm is relatively fast for predicting class labels; it can accurately determine the probabilities for a data point being part of each class with high precision and accuracy. Naive Bayes assumes that all of the features in a collection are independent of each other. The Naive Bayes is trained on labelled DNA sequences where each sequence has its corresponding junction type. The inherent probabilistic nature of DNA bases is exploited to make predictions for unseen DNA sequences. The pseudocode of the training algorithm for the ensemble models used for DNA splice junction prediction is given by Algorithm 2.

**Algorithm 2: The training for the ensemble models**

**Input**: DNA splice junction training fold (*S*_*i*_), maximum number of iterations (*T*),DNA

splice junctions’ features size (L) and population size (N).

**Output**: Trained ensemble model for DNA splice junction prediction, *M*_*i*_.

 **1** Create a random population of wolves that includes *N* candidate solutions, *w*_*i*_, each encoded as a binary vector of length *L*.

 **2** Set t = 1.

 **3** **DO**

 **4**  Calculate the fitness value for each *w*_*i*_ in the population using Eq (2).

 **5**  Set α to the wolf with the best fitness.

 **6**  Set βto the wolf with the second-best fitness.

 **7**  Set Δto the wolf with the third-best fitness.

 **8**  Update the positions of wolves related to α, β, and Δ [31].

 **9**  Binarize wolves’ positions using Eq (1).

 **10**  Set t = t+1.

 **11 While**(t≤T)

 **12** Return the trained ensemble model corresponding to the α wolf, *M*_*i*_.

The following section explains the implementation details for the experiments and the performance evaluation of the proposed ensemble-hybrid approach for DNA splice junction prediction.

## Experiments

This section exhibits the efficacy of the proposed method for predicting the DNA splice junction. The experiments were conducted using the StatLog project’s Primate Splice-Junction Gene Sequences [38]. The StatLog DNA dataset is a processed version of the Irvine database that includes 3186 splice junctions described by 180 binary indicator variables. Each data point is designated as an exon-intron junction (EI), an intron-exon junction (IE), or neither (N). The original 60 symbolic attributes (representing the nucleotides A, G, T, and C) were replaced with three binary indicator variables, each for 180 attributes. The labelled dataset contains 767 data points from the EI class, 765 from the IE class, and 1654 from the N class. The performance of the proposed methodology was evaluated using several experiments. The first experiment used cross-validation with different key fold sizes to assess the model’s generalization capability. The second experiment used a 70%/30% training/test split to estimate the model’s performance on unseen data. In our study, we conducted a series of brute-force preliminary experiments to optimize GWO’s settings and identify suitable control parameters. The algorithm’s performance is evaluated with various parameter combinations. The most promising settings found by these experiments are presented in Table 3. Despite some variation introduced by different chaotic maps, their impact on performance was close.

**Table 3:** GWO control parameters settings.

Parameters	value
Population size	30
Number of iterations	80
Solution dimension	180
a	2.0

The values of these control parameters are fixed for the following experiments. Each experiment is performed ten times to account for the stochasticity of the adopted metaheuristic algorithm and generate robust results, and the mean results are reported. Various metrics are used to evaluate the performance of the proposed methodology. The metrics are computed based on the confusion matrix obtained on the test set. The following terminologies are used to calculate the performance metrics: True positive (TP) and true negative (TN) are the cases where the proposed DNA splice junction prediction model correctly predicts a positive and negative outcome, respectively. False positive (FP) and false negative (FN) are the number of cases where the model incorrectly predicts a positive and negative outcome, respectively. The efficiency of the proposed methodology is evaluated using the following metrics:

- **Accuracy**: The percentage of correct classification, including samples for all DNA splice junction types (EI, IE, or N), is calculated by:

$$Accurecy=\frac{\left(TP+TN\right)}{total\;number\;of\;samples}\times 100$$ (3)

- **TP-rate**: the proportion of actual positive cases that are correctly predicted as EI, IE, or N, is also known as recall; it is calculated by:

$$TP\_rate=\frac{TP}{\left(TP+FN\right)}$$ (4)

- **Precision**: the proportion of positive predictions of splice junction type that are correct; it is calculated by:

$$Precision=\frac{TP}{\left(TP+FP\right)}$$ (5)

- **F-measure**: the accuracy of a DNA splice junction prediction model, taking into account both precision and recall, it is calculated by:

$$F\_measure=2\times \frac{Precision\times Recall}{Precision+Recall}$$ (6)

- **MCC**: Matthews correlation coefficient represents the correlation coefficients between the actual and the predicted class among the DNA splice junction types; it is calculated by:

$$MCC=\frac{TP\times TN-FP\times FN}{\sqrt{\left(TP+FP)\times (TP+FN)\times (TN+FP)\times (TN+FN\right)}}$$ (7)

- **AUC**: Area under curve, it shows the relationship between the actual positive rate and the false positive rate at different thresholds; it is calculated by:

$$AUC=\frac{1}{2}\left(\frac{TP}{\left(TP+FN\right)}+\frac{TN}{\left(TN+FP\right)}\right)$$ (8)

**Fig 4:**
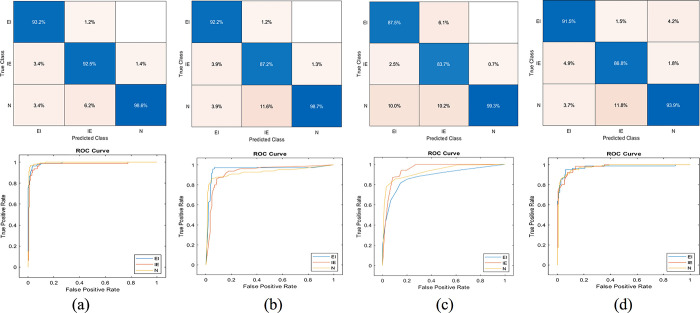
Confusion matrix and ROC curves, tenfold cross-validation experiment. Support vector machine (a), Decision tree (b), k-nearest neighbour (c), Naïve Bayes (d).

**Fig 5:**
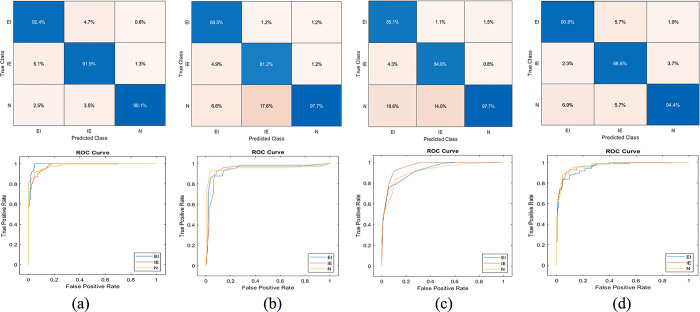
Confusion matrix and ROC curves, fivefold cross-validation experiment. Support vector machine (a), Decision tree (b), k-nearest neighbour (c), Naïve Bayes (d).

**Fig 6:**
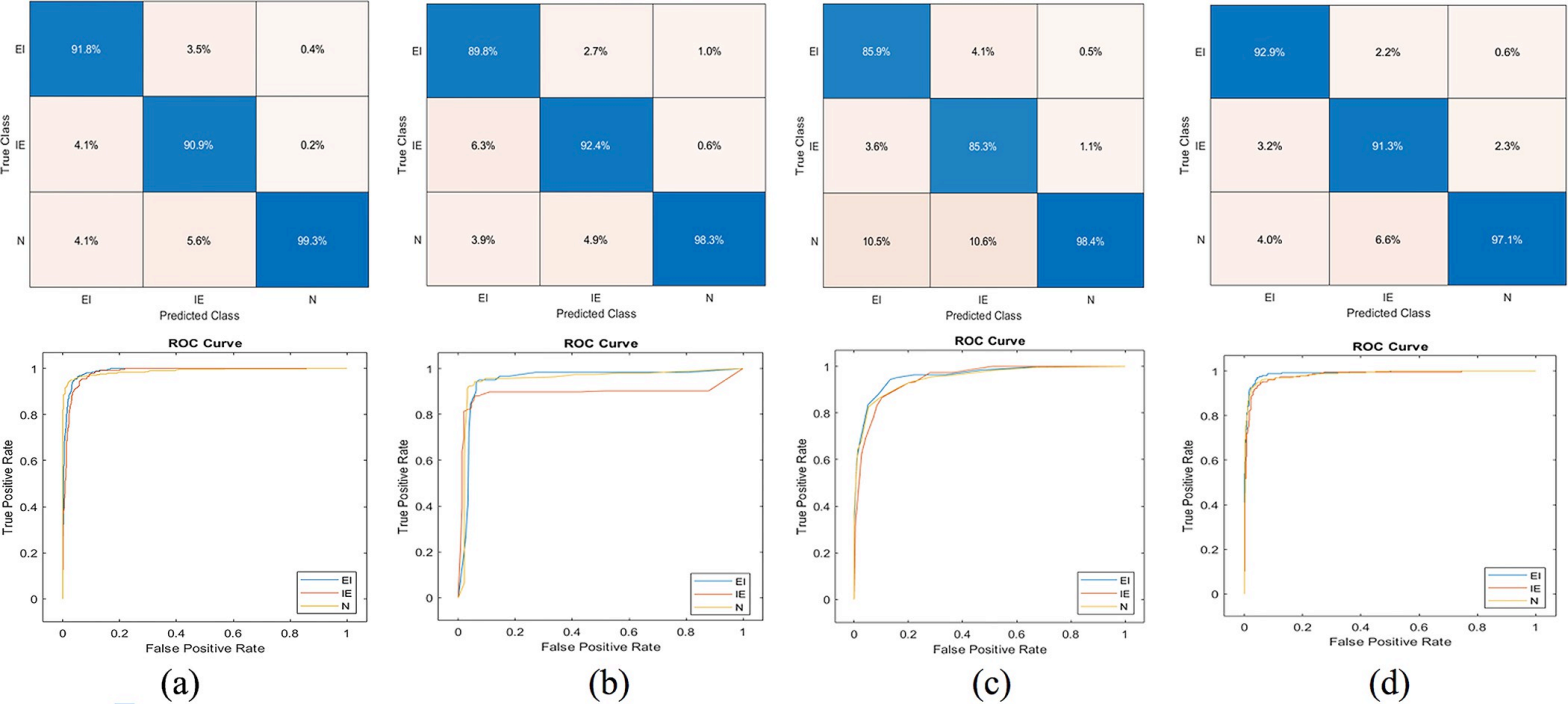
Confusion matrix and ROC curves, 70% training, 30% test experiment. Support vector machine (a), Decision tree (b), k-nearest neighbour (c), Naïve Bayes (d).

In k-fold cross-validation experiments, the dataset is divided into k-equal subsets. Each subset is used as a test set once, while the remaining k-1 subsets are used as the training set. This process is repeated k times, and the average performance of the model across all folds is used as the final evaluation metric. The performance results are shown in Tables 4–6. Moreover, the confusion matrix and ROC curve results are visualized in Figs 4–6.

**Table 4:** Performance results, tenfold cross-validation experiment.

Model	Accuracy	F-measure	MCC	AUC	Classes
GWO+SVM	95.83	0.9711	0.9414	0.9703	N
		0.9366	0.9163	0.9511	IE
		0.9530	0.9384	0.9666	EI
GWO+DT	94.32	0.9619	0.9224	0.9609	N
		0.9169	0.8903	0.9359	IE
		0.9298	0.9087	0.9567	EI
GWO+KNN	89.04	0.9135	0.8408	0.9223	N
		0.8681	0.8285	0.8917	IE
		0.8692	0.8273	0.9024	EI
GWO+NB	93.38	0.9520	0.9007	0.9505	N
		0.9057	0.8761	0.9346	IE
		0.9223	0.8982	0.9490	EI

**Table 5:** Performance results, fivefold cross-validation experiment.

Model	Accuracy	F-measure	MCC	AUC	Classes
GWO+SVM	96.63	0.9736	0.9460	0.9725	N
		0.9385	0.9189	0.9529	IE
		0.9511	0.9359	0.9648	EI
GWO+DT	94.53	0.9553	0.9052	0.9512	N
		0.9021	0.8728	0.9274	IE
		0.9211	0.8979	0.9458	EI
GWO+KNN	90.56	0.9121	0.8380	0.9203	N
		0.8677	0.8243	0.8977	IE
		0.8562	0.8114	0.8863	EI
GWO+NB	95.23	0.9581	0.9148	0.9571	N
		0.9154	0.8902	0.9401	IE
		0.9374	0.9163	0.9563	EI

**Table 6:** Performance results, 70%training, 30% test experiment.

Model	Accuracy	F-measure	MCC	AUC	Classes
GWO+SVM	95.18	0.9711	0.9444	0.9727	N
		0.9272	0.9049	0.9463	IE
		0.9392	0.9165	0.9518	EI
GWO+DT	94.67	0.9701	0.9397	0.9696	N
		0.9196	0.8951	0.9489	IE
		0.9249	0.8997	0.9410	EI
GWO+KNN	91.42	0.9325	0.8748	0.9392	N
		0.8913	0.8575	0.9160	IE
		0.9029	0.8684	0.9205	EI
GWO+NB	94.56	0.9594	0.9187	0.9593	N
		0.9147	0.8879	0.9433	IE
		0.9474	0.9294	0.9586	EI

The results show that the GWO+SVM model outperforms the GWO+DT, GWO+KNN and GWO+NB models on all metrics. The GWO+SVM model achieves an average accuracy of 95.88% across the experiments, while the GWO+DT, GWO+KNN and GWO+NB models achieve average accuracies of 94.50%,90.34% and 94.39%, respectively. The GWO+SVM model also outperforms the other three models on the other metrics, including TP-rate, precision, F-measure, MCC, and AUC. This suggests that the GWO+SVM model can correctly identify positive cases (TP-rate), avoid false positives (precision), and balance the two metrics (F-measure). It also indicates that the GWO+SVM model can distinguish between the different classes better than the other three models. The GWO+SVM model can learn more complex patterns in high-dimensional data of the DNA sequences. Additionally, the optimal feature size obtained by the three experiments using GWO+SVM, GWO+DT, GWO+KNN and GWO+NB are shown in Table 7.

**Table 7:** Optimal feature size for all experiments.

Experiment	GWO+SVM	GWO+DT	GWO+KNN	GWO+NB
*tenfold*	74	63	58	70
*fivefold*	72	63	61	68
*70% training*, *30% test*	74	62	58	71

As shown in Table 7, the average feature size for GWO+SVM was 73, while the average feature size for GWO+DT, GWO+KNN and GWO+NB was 63, 59 and 70, respectively. This indicates that the proposed feature selection layer can search the DNA feature space to find the subset that performs best on the training data. Eventually, Table 8 shows the average fitness values obtained by the proposed hybrid approach.

**Table 8:** Fitness values for all experiments.

Experiment	GWO+SVM	GWO+DT	GWO+KNN	GWO+NB
*tenfold*	0.0363	0.0681	0.0753	0.0573
*fivefold*	0.0263	0.0645	0.0641	0.0337
*70% training*, *30% test*	0.0469	0.0539	0.0491	0.0451

**Fig 7:**
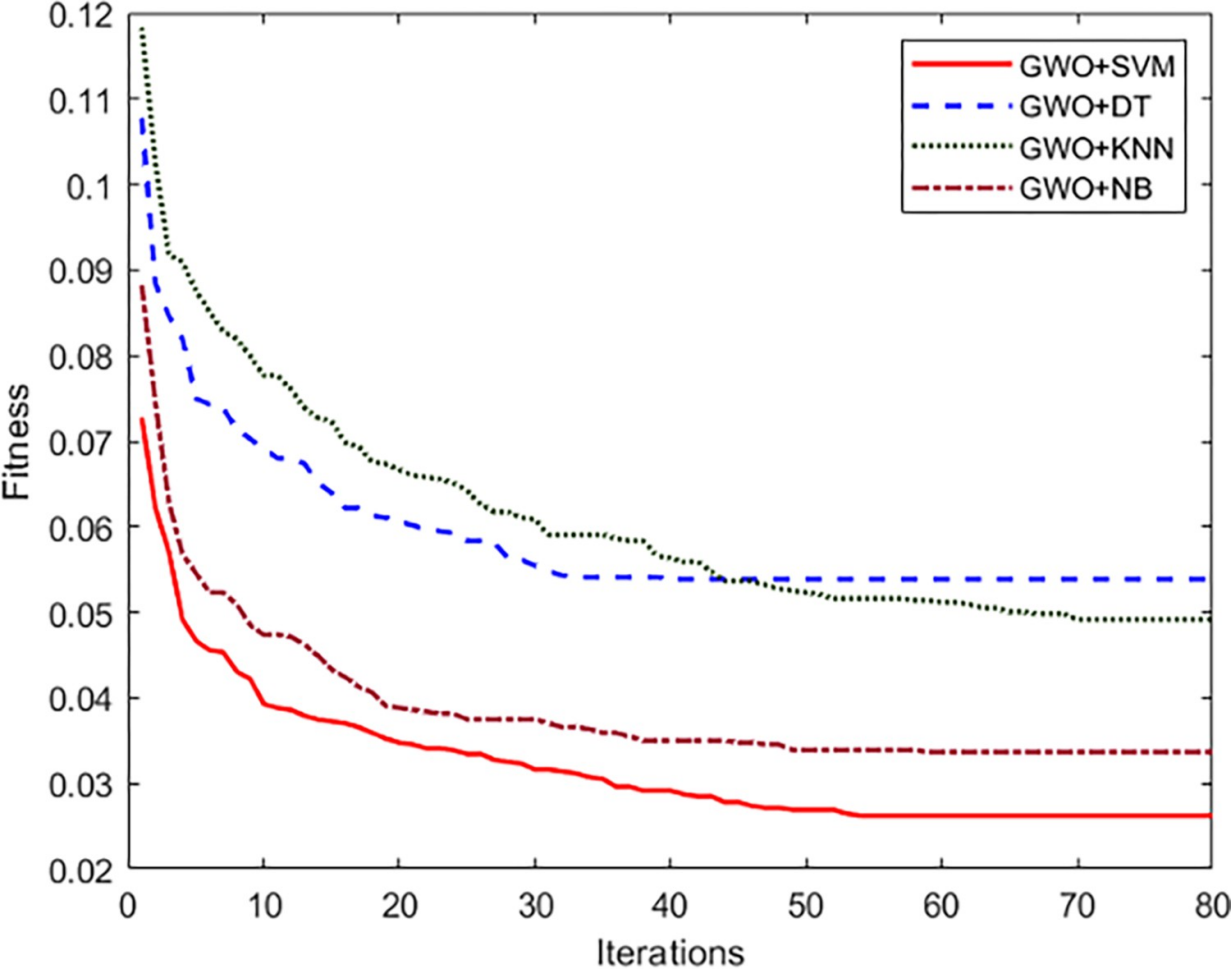
Convergence curves for the applied algorithms.

The results in Table 8 show that GWO+SVM outperformed other classifiers in all three experiments, with the lowest error rate on the training data. This suggests that GWO+SVM is a more robust DNA splice junction prediction algorithm. To further analyze the proposed method, the convergence curves for the applied algorithms are depicted in Fig 7. The curves represent the best fitness across iterations.

The convergence curves in Fig 7 show that all applied algorithms could converge to a good solution. However, GWO+SVM converged the fastest, followed by GWO+NB, GWO+DT and GWO+KNN. The fast convergence is likely because GWO+SVM can learn more complex relationships between the DNA features. Eventually, the proposed method is compared to other DNA splice junction prediction methods in the literature. Table 9 shows the results of this comparison.

**Table 9:** Comparative study analysis.

Ref	Year	Method	Accuracy
[39]	2006	Linear SVM	92.35%
[40]	2010	WPSSM + GA	95.69%
[41]	2013	SOHMMM	91.00%
[11]	2019	CNN	90.25%
[24]	2023	CNN and BLSTM	96.00%
Proposed	-	GWO+SVM ensemble	96.63%

The results in Table 9 show that the proposed GWO+SVM ensemble method achieved a promising accuracy for DNA splice junction prediction compared to the state-of-the-art methods. The proposed hybrid method combines the strengths of two powerful machine learning algorithms: the Grey Wolf Optimizer and the Support Vector Machine.

## Discussion

Within this research work, the two-layer method suggested in this study was evaluated for splicing junction DNA classification. The technique was evaluated on the Primate Splice-Junction Gene Sequences dataset, a part of the StatLog project, and its results were compared with the state-of-the-art methodology. The findings indicate that the proposed ensemble GWO-SVM model can be efficiently employed to predict DNA splicing junction type and has achieved a performance rate of 96.63%. Therefore, this is a significant step because precise DNA splice junction prediction is crucial during gene analysis, disease diagnosis, and bio-drug discovery. The key idea of the GWO+SVM ensemble is the joint utilization of the vital characteristics of the grey wolf optimizer for feature selection and ensemble learning for classification. The proposed ensemble model can choose the features and predict the DNA junction type correctly. This work provides an approach with much potential to be used by researchers and clinicians working in different fields of biology and medicine.

It should be noted that this study used 3186 splice junctions during the experiments, which are available in the Primate Splice-Junction gene sequences. However, future research with more diverse and extensive datasets should be carefully done to assess the designed approach’s applicability. To further advance this research and explore its full potential, evaluating the ensemble approach’s performance with different feature selection methods should be investigated. Developing the approach for analyzing other biological sequences will also be a significant step toward understanding gene regulation.

## Conclusion

This study presents an efficient two-layer hybrid approach based on ensemble learning for DNA splice junction classification. The first layer employs an optimization strategy for feature reduction and selection. The grey wolf optimizer (GWO) is applied to identify the most relevant features from the DNA sequence. On the other hand, the second layer applies an ensemble machine learning model trained on the selected features generated by the previous layer. The cross-validation approach thoroughly evaluates the classifier’s generalization ability, while majority voting assures a solid final decision. The experiments were carried out on Primate Splice-Junction Gene Sequences (DNA) derived from the StatLog project, a processed version of the Irvine database. The proposed method outperforms existing approaches regarding classification accuracy and generalization performance. The results demonstrated that the suggested methodology is more robust to overfitting, highlighting its potential as a promising tool for DNA splice junction classification in computational biology.
